# Prognostic role of plasma fibrinogen in patients with uterine leiomyosarcoma – a multicenter study

**DOI:** 10.1038/s41598-017-13934-8

**Published:** 2017-11-03

**Authors:** Christine Bekos, Christoph Grimm, Thomas Brodowicz, Edgar Petru, Lukas Hefler, Daniel Reimer, Horst Koch, Alexander Reinthaller, Stephan Polterauer, Mariella Polterauer

**Affiliations:** 1 Department of General Gynaecology and Gynaecological Oncology, Gynecologic Cancer Unit - Comprehensive Cancer Center, Medical University of Vienna, Waehringer Guertel 18-20, 1090 Vienna, Austria; 2 Clinical Division of Oncology, Department of Medicine 1, Comprehensive Cancer Center - Medical University Vienna, Waehringer Guertel 18-20, 1090 Vienna, Austria; 3 Department of Obstetrics and Gynaecology of the Medical University of Graz, Graz, Austria; 4 Department of Gynaecology, Barmherzige Schwestern Hospital Linz, Linz, Austria; 5 Department of Obstetrics and Gynaecology of the Medical University of Innsbruck, Tirol, Austria; 6 Department of Obstetrics and Gynaecology of the Medical University of Salzburg, Salzburg, Austria; 7 Karl Landsteiner Institute for General Gynecology and Experimental Gynecologic Oncology, Vienna, Austria

## Abstract

Fibrinogen has an important pathophysiological role in tumor cell progression and development of metastases in different types of cancer. The present study aimed to evaluate the role of pre-treatment fibrinogen plasma concentrations as a biomarker for tumor biology and prognosis in patients with uterine leiomyosarcoma (ULMS). Clinical data of patients with ULMS were assessed in this multi-center study Pre-therapeutic fibrinogen plasma concentrations were evaluated. We investigated the association between fibrinogen plasma levels and clinico-pathological parameters and performed univariate and multivariable survival analyses. In total, 70 women with ULMS were included into the analysis. Mean (SD) pre-treatment fibrinogen plasma levels were 480.2 (172.3) mg/dL. Patients with advanced tumor stage, increased tumor size and higher histological grading had higher fibrinogen levels (p = 0.02, p = 0.013, and p = 0.029, respectively). In ULMS patients with increased fibrinogen levels 5-year overall survival (OS) rates were 25.0% compared to 52.9% in ULMS patients with normal fibrinogen, respectively. Univariate survival analyses revealed that elevated fibrinogen plasma levels (*p* = 0.030), advanced tumor stage (*p* < 0.001) and undifferentiated histology (*p* = 0.003) showed association with unfavorable OS. In multivariable analysis, histological grade (*p* = 0.03) and tumor stage (0.02) were independently associated with survival. Elevated fibrinogen plasma levels were associated with aggressive tumor biology and poor prognosis in women with ULMS. Fibrinogen might be useful as a novel biomarker in ULMS.

## Introduction

Fibrinogen is a plasma glycoprotein rising during systemic inflammation and tissue injury. It plays a leading role in platelet aggregation, clot formation, wound healing, and coagulation^1,2^. Fibrinogen is mainly produced by hepatocytes, but also extrahepatic synthesis by epithelial and tumor cells has been demonstrated^3^.

The association between hemostasis and cancer and the influence of hemostatic factors on cancer development, growth, and metastasis is evident^4,5^. Hypercoagulation in malignant diseases results from the ability of tumor cells to express and release procoagulant factors, such as cancer procoagulant and tissue factor, leading to an activation of the host hemostatic system^6^. Indirect activation may occur through the production of tumor-associated cytokines that trigger tissue factor production by host macrophages or endothelial cells as a host versus tumor response. Furthermore, impaired fibrinolysis and lower levels of coagulation inhibitors contribute to the hypercoagulated state^7,8^. Fibrin, fibrinogen, and other coagulation factors play an active role in tumor cell growth, invasion, and metastasis by supporting the sustained adhesion of tumor cells and promoting tumor neoangiogenesis via fibroblast growth factor (FGF)-2^9–11^.

Fibrinogen is also one of the major acute phase proteins, synthesized during inflammation and stress^12,13^. It has been shown that tumor development and growth of various tumors, including uterine cancers, are closely associated to inflammatory processes^4,5^. The inflammatory microenvironment of tumors is part of the neoplastic process and promotes proliferation, survival, and migration of tumor cells^4,14^. Furthermore, fibrinogen itself can directly bind to inflammatory or tumor cells leading to induced synthesis of proinflammatory cytokines^15^.

Neoplastic cells make use of several signaling molecules of the innate immune system for invasion, migration, and metastasis^6,16^. In recent publications plasma fibrinogen levels were shown to be useful as prognostic parameter for different gynecologic malignancies, such as endometrial, cervical, vulvar and ovarian cancer^17–20^. In addition, plasma fibrinogen was shown to be a valid prognostic parameter in patients with soft tissue sarcoma and malignant tumors showed higher fibrinogen levels when compared to benign soft tissue tumors^21,22^. For ULMS valid prognostic biomarkers are sparse. Recently markers such as CRP, GGT and CA 125 have been investigated for assessing prognosis in women with ULMS^23–25^.

In the present study we aimed to evaluate the role of pre-therapeutic fibrinogen concentrations in tumor characteristics and prognosis of patients with ULMS.

## Results

Patients’ demographics are shown in Table 1.

**Table 1:** Characteristics of 70 patients with uterine leiomyosarcoma.

Variable	N (%) or Mean (SD)
**Patients**	70
**Age** (**years**)	52.4 (10.6)
**Pretherapeutic Fibrinogen** (**mg/dl**)	480.2 (172.3)
**Tumor stage**	
FIGO IA	13 (18.6)
FIGO IB	21 (30.0)
FIGO II	5 (7.1)
FIGO III	6 (8.6)
FIGO IV	25 (35.7)
**Tumor size** (**cm**)	
<5	8 (11.4)
5–10	23 (32.9)
>10	30 (42.9)
Unknown	9 (12.9)
**Histological Grading**	
G1	5 (7.1)
G2	8 (11.4)
G3	43 (61.4)
Unknown	14 (20.0)
**Primary Metastatic Site**	
Lymph nodes	7 (10.0)
Lungs	17 (24.3)
Bone	4 (5.7)
**Status at last follow up**	
Alive	25 (35.7)
Dead	45 (64.3)
**Follow Up Time** (**months**)*	30.0 (1–204)

FIGO: International Federation of Gynaecology and Obstetrics.

In the present study, mean (SD) pre-treatment fibrinogen plasma values were 480.2 (172.3) mg/dL. Mean fibrinogen values broken down by clinico-pathological parameters are provided in Table 2.

**Table 2:** Fibrinogen values broken down by clinico-pathological parameters of 70 patients with uterine leiomyosarcoma.

**Parameter**	**Fibrinogen (mg/dL) (SD)**	**P-value**
**Age (years)**		0.56^a^
<**52.4**	438.6 (167.6)	
≥**52.4**	517.3 (170.1)	
**Tumorstage**		0.02^b^
**FIGO IA**	371.9 (159.2)	
**FIGO IB**	446.1 (155.9)	
**FIGO II**	519.6 (83.9)	
**FIGO III**	599.9 (274.9)	
**FIGO IV**	528.4 (147.7)	
**Tumorsize (cm)**		0.013^b^
**<5**	349.1 (97.8)	
**5–10**	466.5 (169.7)	
**>10**	545.4 (171.3)	
**Grading**		0.029^b^
**G1**	412.0 (157.8)	
**G2**	494.0 (224.0)	
**G3**	520.8 (162.0)	

FIGO: International Federation of Gynaecology and Obstetrics.

^a^Students’ t-test, ^b^One-way ANOVA.

Patients with advanced tumor stage, increased tumor size and higher histological grading had higher fibrinogen levels (*p* = 0.02, p = 0.013 and p = 0.029, respectively).

In ULMS patients with increased fibrinogen compared to patients with normal fibrinogen levels, 5-year overall survival (OS) rates were 25.0% and 52.9%, respectively. All of these patients had no signs of acute infection or inflammatory process at time of diagnosis and blood draw. Univariate survival analyses revealed that elevated fibrinogen plasma levels (*p* = 0.03), advanced tumor stage (*p* < 0.001) and undifferentiated histology (*p* = 0.003) showed association with poor OS. Results of the univariate Kaplan-Meier analysis and the multivariable Cox regression model are shown in Table 3. Patients’ demographics of women with ULMS and normal concentrations of Fibrinogen are shown in Table 4.

**Table 3:** Survival analysis of 70 patients with uterine leiomyosarcoma.

Parameter	Univariate		Multivariable	
	5-year OS	p-value	HR (95% CI)	p-value
**Fibrinogen**		0.03	1.3 (0.60–2.83)	0.51
<400 mg/dl	52.9%			
>400 mg/dl	25.0%			
**Age**		0.68	0.6 (0.34–1.24)	0.19
<2.4	36.4%			
>52.4	31.5%			
**Tumor stage**		<0.001	1.3 (1.05–1.70)	0.02
FIGO IA	81.5%			
FIGO IB	43.4%			
FIGO II	20.0%			
FIGO III	33.3%			
FIGO IV	20.5%			
**Grading**		0.003	1.5 (1.04–2.05)	0.03
G1	100%			
G2	43.8%			
G3	31.1%			

OS: overall survival, HR: hazard ratio, CI: confidence interval.

**Table 4:** Demographic characteristics of 26 patients with uterine leiomyosarcoma and fibrinogen within the normal range.

Variable	N (%) or Mean (SD)
**Age at diagnosis** (**years**)	49.7 (11,4)
**Pretherapeutic Fibrinogen** (**mg/dl**)	312.5 (99.7)
**Tumor stage**	
FIGO IA	8 (30.8)
FIGO IB	9 (34.6)
FIGO II	1 (3.8)
FIGO III	2 (7.7)
FIGO IV	6 (23.1)
**Tumor size** (**cm**)	
<5	6 (23.1)
5–10	9 (34.6)
>10	7 (26.9)
Unknown	4 (15.4)
**Histological Grading**	
G1	3 (11.5)
G2	5 (19.2)
G3	10 (38.5)
Unknown	8 (30.8)

**Figure 1:**
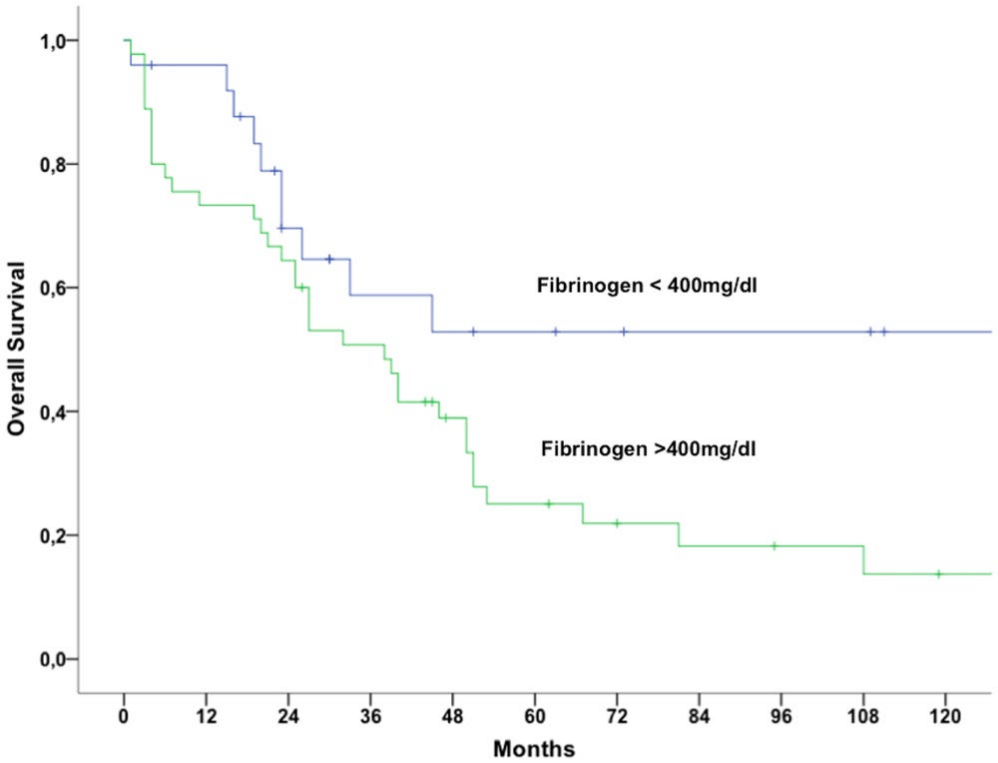
Kaplan-Meier survival analysis in patients with uterine leiomyosarcoma distributed by pre-therapeutic fibrinogen levels.

Women with elevated fibrinogen plasma concentrations present with unfavorable OS compared to patients with fibrinogen within the normal range. In Fig. 1, Kaplan-Meier survival curves show the association between pre-therapeutic fibrinogen levels and OS. In multivariable analysis, undifferentiated histology and tumor stage were showed association with poor prognosis (*p* = 0.03 and p = 0.02, respectively).

## Discussion

In this multicenter study, elevated pretherapeutic plasma fibrinogen levels were demonstrated to show association with poor prognosis in the studied population with ULMS. This is the first study investigating the clinico-pathological and prognostic value of pretreatment plasma fibrinogen in patients suffering from ULMS.

Our investigations are in concordance with previously reported data, that showed that plasma fibrinogen can be used as prognostic parameter in various solid tumors such as endometrial, cervical, vulvar and ovarian cancer as well as in soft tissue sarcoma (STS)^17–20,22,26^. ULMS is defined as a subtype of STS. Szkandera *et al*. reported of significantly reduced OS in patients with STS if pre-therapeutic fibrinogen plasma levels were increased^21^. However, the group of STS includes several different tumor types^27,28^. The present analysis is the first report including exclusively patients with ULMS.

Previous reports showed that elevated fibrinogen plasma levels can be caused either by a state of hypercoagulation and hypoxia caused by growing tumors, by production of tumor cells themselves, or by inflammation mediating cells such as epithelial cells as a host versus tumor response^27^. Furthermore, it has been demonstrated that fibrinogen itself actively modulates the inflammatory process by inducing synthesis of proinflammatory cytokines from peripheral blood mononuclear cells and by interacting with leukocytes^4,5^. Overall, a chronic inflammatory process is regarded vital for tumor initiation, invasion, and formation of metastases but it is unclear to date, which of these mechanisms are predominant.

In line with other studies in patients with ULMS^21^, tumor stage, and histologic grading had a strong impact on OS in our patient cohort. Finally, grading and tumor stage were the only two parameters that were independently associated with overall survival in multivariable analysis. It has to be noted that information on histological grade was not available for all patients of the cohort. The missing values might have influenced the results of the multivariable analysis with respect to fibrinogen showing no significance.

Increased pre-therapeutic plasma fibrinogen levels were associated with higher tumor stage, increased tumor size and higher histological grading. This is in line with previously published data in patients with other malignant diseases^17–20,29,30^, supporting the assumption that plasma fibrinogen seems to increase with tumor growth, progression, and metastasis. In a transgenic mouse model it has been demonstrated that fibrinogen plays a major role in the development of metastases but not in the growth of the primary tumor^9^. This is consistent with our data showing increased fibrinogen levels in tumor stages III and IV with lymphogenic and distant metastases compared to stages I and II. This hyperfibrinogenemia in advanced stages might be explained by endogenous fibrinogen production by tumor cells^3^. Another line of evidence suggests induction by an inflammatory reaction to tumor growth as a source for increased fibrinogen^6^. The production of tumor-associated cytokines that trigger tissue factor production by host macrophages or endothelial cells could be an explanation for this indirect activation^5^.

We observed that plasma fibrinogen levels were significantly elevated in high-grade tumor histology. High-grade tumors were associated with unfavorable prognosis and lower 5-year survival rates. Therefore, it seems that plasma fibrinogen is not only a marker for advanced disease but also for aggressive histology and tumor biology. Furthermore high-grade tumors are associated with more necrotic tumor cells, which might promote “inflammation”. Similar findings were reported for other gynecologic cancers such as vulvar and endometrial cancer as well as for soft tissue sarcomas^19–21^. In a study investigating STS, classified in 18 different histologies, pretreatment plasma fibrinogen levels were associated with cancer-specific survival, disease-free survival and overall survival in patients with tumor ≥5 cm, grade 3 and high-risk features^21^. It is interesting that fibrinogen plasma concentrations did not correlate with patients’ age, since there is evidence that other acute phase proteins are elevated in older patients. However, similar results were found in another recent study with ULMS patients^25^.

Besides the fact that the plasma fibrinogen level reflects the activation of blood coagulation, fibrinogen has been demonstrated to actively modulate the inflammatory process by synthesizing pro-inflammatory cytokines from peripheral blood mononuclear cells and by interacting with leukocytes^15,31^. In a preclinical model, fibrinogen itself has been identified as a critical determinant for altering tumor cell metastatic potential in lung carcinoma and melanoma. Lower tumor cell survival and reduced metastatic potential was observed in fibrinogen-deficient mice^16^. These findings established an important link between hemostatic factors and innate immunity. This might indicate that one mechanism by which the platelet–fibrinogen axis contributes to metastatic potential is by preventing adherence of natural killer cell, monocytes and lymphocytes to tumor cells. Platelet–fibrin micro thrombi seem to act as a physical barrier avoiding tumor cell elimination^11^.

When interpreting this study several potential limitations should be taken in account. This is a retrospective trial comprising patients from a rather long treatment interval and different centers. The management of patients with ULMS has been modified through the years and the impact of these changes were beyond the scope of this retrospective study. Additionally, the number of subjects participating in this study is of course limited and therefore multivariable analysis might have been influenced negatively. Apart from that, ULMS belong to the group of orphan diseases and this is the first trial that investigated the role of pre-treatment fibrinogen plasma concentrations as prognostic factor in this malignant disease. Plasma fibrinogen level is an established laboratory test that is utilized in daily clinical routine and is relatively cheap. Previously only few serum biomarkers were shown to have prognostic value in women with ULMS^23–25^. Therefore we think that the identification of fibrinogen as a potential novel marker is clinically and scientifically relevant.

Our results showed an association between elevated plasma fibrinogen levels and unfavorable overall survival in patients with ULMS in a univariate survival analysis. However, the present results need to be confirmed in further studies. Fibrinogen might then be used for individual risk stratification and patient counseling. In particular in patients with ULMS were reliable prognostic biomarker are missing, fibrinogen could be of additional clinical value.

## Materials and Methods

### Patients

Seventy consecutive patients diagnosed with ULMS were treated and enrolled in the present study between 1996 and 2015 at the Comprehensive Cancer Center Vienna, Vienna, Department of Obstetrics and Gynecology of the Medical University of Graz, Styria, and the Department of Gynecology, Barmherzige Schwestern Hospital Linz, Upper Austria, Austria, Department of Obstetrics and Gynecology of the Medical University of Innsbruck, Tirol, Department of Obstetrics and Gynecology of the Medical University of Salzburg, Salzburg. Clinical data were obtained using available tumor databases and by electronic chart review. The 2009 International Federation of Gynecology and Obstetrics (FIGO) classification system was used^32^. Clinical examination, magnetic resonance imaging (MRI) and/or computed tomography (CT) were performed for primary tumor assessment. The management of these patients consisted of surgical staging including hysterectomy, bilateral salpingo-oophorectomy, pelvic and/or paraaortic lymphadenectomy if enlarged lymph nodes were palpable and cytoreductive procedure in women with extra-uterine disease as described previously^33^. If clinically indicated, radiation therapy, adjuvant and/or palliative chemotherapy was given based on physician choice. Before starting the therapy physical examination by a specialist in internal medicine was conducted and patients with signs of inflammation were excluded from the study. Follow-up included clinical examinations and radiologic studies when indicated and was performed every three months for the first three years. From the third to the fifth year patients were evaluated every six months. Afterwards check-ups were performed annually up to ten years.

The study was approved by the Ethics Committee of the Medical University of Vienna (IRB approval number: 1520/2012) before the study was initiated.

All patients gave their consent to treatment according to institutional guidelines, and all patients had consented to anonymized data extraction and analysis. Since this study was a retrospective analysis the ethics committee waived the requirement to obtain distinct informed consent from patients. The database with patients records was anonymized and de-identified prior to analysis. This study was performed in accordance to the ICH Harmonized Tripartite Guideline for Good Clinical Practice, the Declaration of Helsinki and the guidelines of the Ethics Committee of the Medical University of Vienna.

### Fibrinogen Measurement

Blood samples (citrated plasma) for evaluation of plasma fibrinogen levels were taken by peripheral venous puncture 24–48 hours before starting the treatment. Clotting reagents from Diagnostica Stago (Asnieres, France) were used to determine plasma fibrinogen levels by the Clauss method^34^. The manufacturer claims an intra-assay variability of 3.5%. The normal range for plasma fibrinogen levels is defined between 180 and 390 mg/dl.

### Statistical Analysis

Values are presented as mean values with standard deviation (SD). In order to compare mean fibrinogen plasma concentrations and clinico-pathological findings students’ T- tests and one-way ANOVA tests were performed. P-values of < 0.05 were considered statistically significant. Differences between groups were tested using the log-rank test. The results were analyzed for the endpoint of overall survival (OS). Univariate survival analyses were performed using log-rank test and Cox Regression analyses. For overall 5-year survival, Kaplan-Meier survival curves were calculated. Multivariable analysis was performed using Cox regression including as independent variables fibrinogen (dichotomized at 400.0 mg/dl, indicating elevated values) and patients’ age (dichotomized at the median value of 52.4 years), tumor stage (FIGO IV *vs*. FIGO III *vs*. FIGO II *vs*. FIGO IB *vs*. FIGO IA), and tumor grading (G3 *vs*. G2 *vs*. G1). Statistical analysis was performed using the statistical software SPSS 24.0 for MAC (SPSS 24.0, IBM Inc., Armonk, NY).

The datasets generated during and/or analysed during the current study are available from the corresponding author on reasonable request.
